# Hepatic Pseudolipoma: A Rare Case

**DOI:** 10.7759/cureus.18712

**Published:** 2021-10-12

**Authors:** Mazen Esmaeil, Ahmed K Ahmed, Ahamed M Elkhair, Islam Ahmed, Shamim Haithrous

**Affiliations:** 1 Acute Medicine, Countess of Chester Hospital, Chester, GBR; 2 Radiology, Countess of Chester Hospital, Chester, GBR; 3 Neurology, Mayo Clinic, Jacksonville, USA; 4 Internal Medicine, Medway Maritime, Gillingham, GBR

**Keywords:** anatomy, gastroentero-hepatology, radiology, liver, pseudolipoma

## Abstract

Pseudolipoma, also known as pseudolipoma of the Glisson's capsule, is an encapsulated lesion that contains degenerated fat and is enveloped by the liver capsule. In this report, we discuss a 37-year-old male presenting with dysuria and microscopic hematuria who revealed an incidental finding of a pseudolipoma on a CT scan of the abdomen.

## Introduction

Pseudolipoma, also known as pseudolipoma of the Glisson's capsule, is a mass of fat tissue encapsulated and enveloped by the liver [1]. It originates from part of the epiploic appendix that degenerates and later calcifies, situating between the diaphragm and the liver [2]. Few cases have been reported with a prevalence of 0.2% and a mean age of 67 [3]. Other causes may include traumatic inclusion of fat within the liver capsule during surgery, or by transcutaneous liver biopsy [4]. The liver surface usually consists of a well-circumscribed nodule with a center of fat or soft-tissue attenuation that is highlighted on CT [1]. Patients with hepatic pseudolipoma usually have a history of previous abdominal surgery [5]. Treatment is generally not indicated for hepatic pseudolipoma as it is usually asymptomatic [6].

## Case presentation

**Figure 1:**
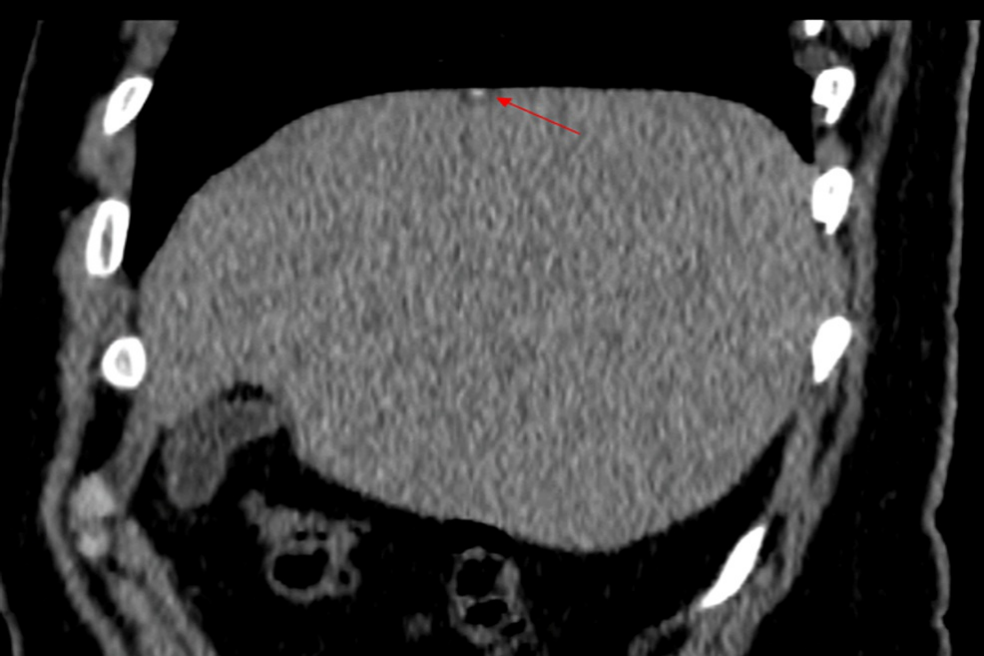
Sagittal CT: cystic lesions within the liver dome with a fleck of calcification.

A 37-year-old male presented due to dysuria and microscopic hematuria which was identified on urine analysis. Computed tomography of kidneys, ureters and bladder (CT KUB) showed no evidence of hydronephrosis or urolithiasis. An incidental cyst was identified within the liver dome (segment VIII), with flecks of calcification. Otherwise, the CT appearance of the liver parenchyma was normal with no other focal lesions (Figure 1). Cystoscopy and CT urogram showed normal urinary tracts, and empirical treatment with antibiotics resolved symptoms. Renal function tests, liver function tests, and inflammatory markers were all normal. 

**Figure 2:**
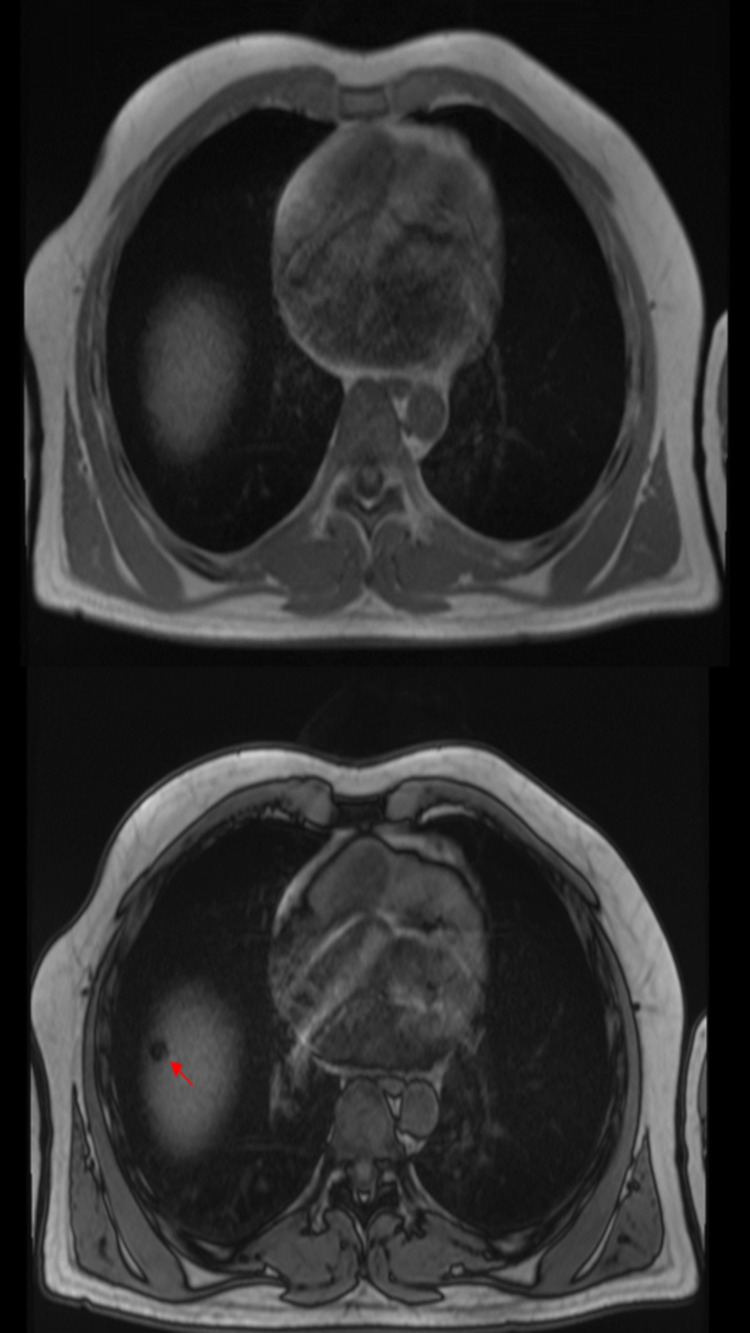
In and out of phase T1: shows signal drop out in the out of phase imaging indicating fat.

**Figure 3:**
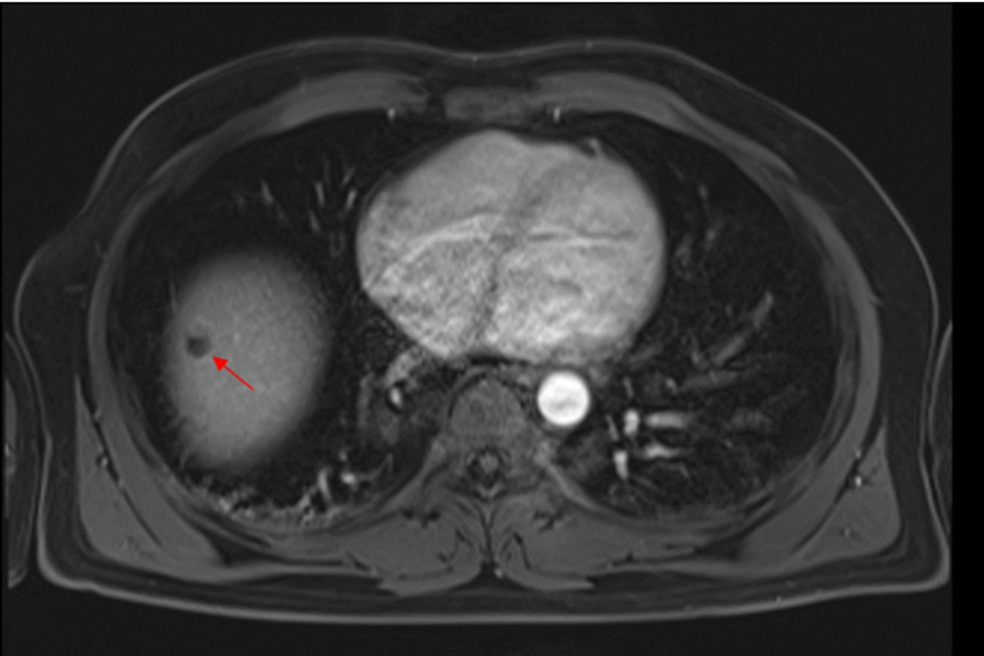
Arterial enhanced T1: no arterial enhancement shown within the lesion.

An MRI was done to further characterize the liver lesion, and this showed a cystic lesion which was isointense on T2 with complete signal drop out in the out of phase and fat saturated T1 images. The T1 hyperintensity is believed to be artifactual secondary to the calcification seen on CT (Figure 2). Diffusion weighted imaging showed no restriction diffusion (Figure 3). 

The case was discussed at the upper gastrointestinal tract multidisciplinary team meeting, and the consensus was that this represents a benign pseudolipoma of Glisson's capsule.

## Discussion

The liver consists of an outer serous layer, covering the majority of the liver, and an inner layer called the Glisson's capsule. The serous layer does not cover the bare area, porta hepatis, and the attachment site of the gallbladder to the liver. The Glisson's capsule, on the other hand, encapsulates the entire liver. This anatomic features along with the physiologic functions of the liver, exposes the liver capsule to a multitude of complications and disease processes [7]. Pseudolipomas of Glisson's capsule are rare lesions that are usually seen in men of older age (mean age of 67) [6, 8]. However, in this case the patient’s age is 37 years, thus the presentation becomes atypical. 

Pseudolipomas are masses of degenerating fat that encapsulate and adhere to the liver, usually in the subcapsular space. They are thought to be the result of either receding hepatic lipomas or epiploic appendages [9]. On pathological examination, pseudolipomas appear identical to epiploic appendages [6]. On CT, they appear as well-circumscribed nodules on the surface of the liver with fat attenuation [10]. Patients usually demonstrate no symptoms, however, abdominal pain similar to appendicitis or diverticulitis may occur [6]. There is minimal evidence to show whether past histories of abdominal surgery are risk factors in the development of these lesions [6, 8].

Our patient is 37 years old and presented with dysuria and microscopic hematuria. Inflammatory markers and renal function were normal. CT KUB shows no hydronephrosis, however, an incidental cyst was identified within the liver dome (segment VIII), with flecks of calcification. Cystoscopy and CT urogram were normal and the patient’s symptom improved after a course of antibiotic.

An MRI was done to know more about the nature of the liver lesion. The T1 hyperintensity is believed to be artifactual secondary to the calcification seen on CT. Diffusion weighted imaging showed no restriction diffusion while T2 showed a cystic lesion which was isointense on T2 with complete signal drop out in the out-of-phase and fat saturated T1 images.

Further discussion by upper gastrointestinal tract multidisciplinary team meeting agreed that this represents a benign pseudo lipoma of Glisson's capsule.

## Conclusions

Pseudolipoma of Glisson's capsule are benign lesions composed of degenerating fat, likely derived from a dislodged epiploic appendage. Affected patients usually show no symptoms, however, abdominal pain similar to appendicitis or diverticulitis may occur. There is minimal evidence to show whether a patient's past histories of abdominal surgery are risk factors in the development of these lesions.
